# Cardiovascular disease markers in women with polycystic ovary syndrome with emphasis on asymmetric dimethylarginine and homocysteine

**DOI:** 10.4103/0256-4947.65255

**Published:** 2010

**Authors:** Ahmed M. Mohamadin, Fawzia A. Habib, Abdulrahman A. Al-Saggaf

**Affiliations:** From the a Department of Obstetrics and Gynecology, Clinical Biochemistry, College of Medicine, Taibah University, Saudi Arabia; b Department of Obstetrics and Gynecology, Clinical Biochemistry, College of Medicine, Taibah University, Saudi Arabia

## Abstract

**BACKGROUND AND OBJECTIVES:**

Polycystic ovary syndrome (PCOS) is a disorder characterized by hyperandrogenism, ovulatory dysfunction, and polycystic ovaries. Little is known about cardiovascular risk factors in patients with PCOS. We investigated plasma markers of cardiovascular disease in Saudi women with PCOS, with an emphasis on asymmetric dimethylarginine (ADMA) and total homocysteine (tHcy).

**PATIENTS AND METHODS:**

Fifty Saudi women with PCOS diagnosed by the Rotterdam criteria (mean age [SD] 30.2 [3.0] years) and 40 controls without PCOS (mean age 29.3 [2.5] years) had measyrements taken of clinical, metabolic, and hormonal parameters, including plasma ADMA, tHcy, lipoprotein (a) ([Lp(a)], and serum high sensitivity C-reactive protein (hs-CRP), nitric oxid, and fibrinogen. Insulin resistance was calculated by the homeostasis model assessment (HOMA-IR).

**RESULTS:**

Women with PCOS had significantly higher fasting insulin, HOMA-IR, and luteinizing hormone (LH) levels than healthy controls (*P*<.001). Lipid profile, free androgen index (FAI), ADMA, tHcy, hsCRP, and Lp(a) were significantly higher in women with PCOS compared with healthy controls (*P*<.001). The women with PCOS had significantly lower nitric oxide and high-density lipoprotein cholesterol (HDL-C) levels compared with healthy controls (*P*<.001).

**CONCLUSION:**

Our study revealed that Saudi women with PCOS had a significantly different levels of plasma markers of cardiovascular disease compared with normal controls. Therefore, clinicians who manage women with PCOS should follow up on these markers to reduce the risk of cardiovascular disease.

Polycystic ovary syndrome (PCOS) is the most common endocrine disorder of women of reproductive age and is associated with long-term health risks, including type 2 diabetes mellitus (T2DM) and coronary artery disease.^1^,^2^ Most women with PCOS also exhibit features of the metabolic syndrome, including insulin resistance (IR), obesity, and dyslipidemia.^3^,^4^ In 2001, Paradisi et al^5^ first reported that PCOS is characterized by endothelial dysfunction and IR. IR, hyperandrogenism, and dyslipidemia are likely to be the major risk factors for cardiovascular disease (CVD) in women with PCOS.^6^,^7^

Several biochemical markers have been identified as risk factors for CVD, including elevated levels of serum low-density lipoprotein cholesterol (LDL-C), total cholesterol, triglycerides (TG), total homocysteine (tHcy), high-sensitivity C-reactive protein (hsCRP), and IR, as well as reduced levels of high density lipoprotein cholesterol (HDL-C). Decreased nitric oxide bioavailability and hyperhomocysteinemia also increase the risk of CVD.^6^

Asymmetric dimethylarginine (ADMA) is an endogenous inhibitor of nitric oxide synthase.^8^ ADMA is considered an indicator for endothelial dysfunction^9^ and a sensitive marker for cardiovascular risk.^10^,^11^ An inverse correlation between endothelium-dependent vasodilatation and ADMA has also been demonstrated after fat intake and experimental hyperhomocysteinemia in humans.^9^ Hyperhomocysteinemia is another important risk factor for the development of CVD.^12^ Homocysteine is a sulfur-containing amino acid formed during the metabolism of methionine.^11^ It has been reported that high insulin levels are associated with increased plasma levels of tHcy in patients with preeclampsia.^13^

Also, hsCRP has been shown to be a good predictor of vascular events.^14^ In addition to being a marker of inflammation, there is evidence that hsCRP may have a direct role in atherogenesis via adhesion molecule expression, complement activation, and mediation of LDL uptake by macrophages.^15^ Similarly, raised fibrinogen concentration has been associated with an increased risk of ischemic heart disease and atherosclerosis. Fibrinogen may promote CVD by a variety of mechanisms, including increased blood viscosity, thrombus formation, or platelet aggregation.^14^ The aim of the present study was to investigate the plasma markers of CVD in Saudi women with PCOS. Special emphasis was given to ADMA and homocysteine.

## PATIENTS AND METHODS

The study group comprised 50 Saudi women (aged 24 to 31 years) with primary infertility, who had been diagnosed as having PCOS (study group, aged 24 to 31 years) and were attending the outpatients infertility clinic at the Department of Obstetrics and Gynecology of Safa Al-Madinah Hospital, Al-Madinah, Saudi Arabia, from September 2008 to March 2009. Also, 40 healthy Saudi women volunteers (control group, aged 25 to 30 years) formed the control group in for this study. Women in the control group were healthy volunteers. The diagnosis of PCOS was made according to the Rotterdam European Society of Human Reproduction and Embryology (ESHRE)/American Society for Reproductive Medicine (ASRM)-sponsored PCOS Consensus Workshop Group guidelines.^16^ Exclusion criteria included hypertension, smoking, or endocrine disorders and those taking fertility drugs, oral anti-diabetic agents, or oral contraceptive pills, and endocrine disorders.

Venous blood samples were collected from an antecubital vein between 08:00 AM and 09:00 AM after an overnight fast. The samples were centrifuged, aliquoted and immediately frozen and stored at –80°C for biochemical analysis. A full physical examination was performed, including measurement of weight, height and waist and hip circumferences. Weight was measured with the subject wearing light clothing without shoes, and height was measured using a stadiometer. Body mass index (BMI) was calculated by using the formula: weight (in kg)/height (in meters).^2^ Waist circumference (WC) was measured with the patient standing, at a point midway between the lower costal margin and the iliac crest in the mid-axillary line. Blood pressure was measured manually with a sphygmomanometer. Before onset of the study, all women had a full physical examination and were asked to complete a general questionnaire. The study was approved by the Institutional Review Board of the Taibah University and supported partially by funds from the Deanship of Scientific Research (Project No. 73/427) in Taibah University, Al-Madinah, Saudi Arabia. Written informed consent was obtained from all women at the study entry.

Plasma glucose, total cholesterol, triglycerides (TG), HDL-C, and LDL-C levels were measured by the enzymatic method by using the specific commercial kits (Roche/Hitachi 902; Roche Diagnostics GmbH, Manheim, Germany). Plasma insulin, luteinizing hormone (LH), follicle stimulating hormone (FSH), sex hormone-binding globulin (SHBG), 17-hydroxy progesterone (17-OHP), and total testosterone, were determined by enzyme-linked immunosorbent Assay assay (ELISA) kits (R and D Systems, Minneapolis, USA). The concentration of Lp(a) was determined by immunoturbidometry with the Roche instrument and the Cobas Miro Mira Analyzer (Basel, Switzerland). The concentration of fibrinogen was determined using an immunoenzymatic set of markers (Sysmex CA540, Dade-Behring, Germany). The hsCRP concentration was determined using an immunoturbidimetric method (Randox, Mauguio, France). Plasma ADMA and tHcy concentrations were determined by competitive ELISA assay (DLD Diagnostica GmbH, Hamburg, Germany). Nitric oxide concentration was measured using a nitrite/nitrate colorimetric assay kit (Griess reaction, Cat No. 780001; Cayman Chemical, Ann Arbor, MI). All chemicals were of the highest available purity available and were purchased from Sigma-Aldrich Co. (St Louis, MO, USA). The free androgen index (FAI)17 and HOMA-IR^18^ were calculated.

All statistical analysis was performed using the SPSS version 13.0 (SPSS Inc., Chicago, IL, USA). The *t* test was used for comparison of means. Data are expressed as mean and standard error of the mean. *P* values <.05 were considered statistically significant.

## RESULTS

**Figure 1:**
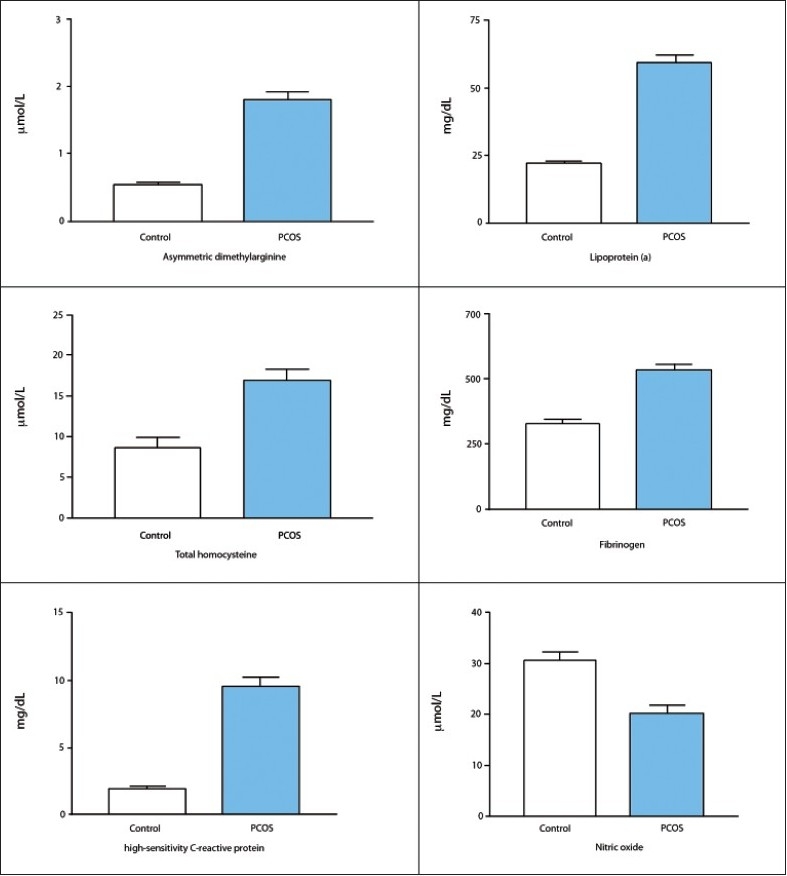
Plasma markers of cardiovascular disease in Saudi women with PCOS vs controls. All differences statistically significant (*P*<.001)

There were no significant differences in age, weight, height, body mass index (BMI), and systolic and diastolic blood pressures (BP) between the PCOS and control groups (Table 1). BMI was lower in healthy controls than in patients with PCOS, but these this differences did not achieve statistical significance. Compared with healthy women, patients with PCOS had significantly higher levels of fasting insulin, HOMA-IR, triglycerides, total cholesterol, LDL-C, and VLDL-C (*P*<.001) (Table 2). On the other hand, patients with PCOS had significantly lower levels of HDL-C (*P*<.001) and a lower glucose/insulin ratio compared with subjects in the control group (Table 2). In PCOS women, total testosterone, LH, LH/FSH ratio, and 17-OHP were significantly increased (*P*<.001) compared with those values in the control group. As expected, FSH was lower in PCOS women than in the control group, but these differences did not achieve statistical significance. Plasma SHBG level was also significantly lower in the PCOS patients group compared with control group. Free androgen index was significantly higher in the PCOS group (6.63%) compared with the control group (2.08%). Patients with PCOS had significantly higher concentrations of ADMA, tHcy, hsCRP, Lp(a), and fibrinogen (Figure 1). Nitric oxide was significantly lower in patients with PCOS.

**Table 1:** Clinical features of women with polycystic ovary syndrome and healthy controls.

Variables	Control (n=40)	PCOS (n=50)	*P* value
Age (years)	29.3 (2.5)	30.2 (3.0)	.412
Weight (kg)	57.8 (3.0)	61.3 (3.3)	.222
Height (cm)	162.3 (6.4)	166.7 (4.6)	.284
Waist circumference (cm)	87.3 (5.0)	93.5 (7.0)	.246
Hip circumference (cm)	105.0 (8.0)	117.0 (10)	.184
Waist/Hip ratio	0.83 (0.05)	0.79 (0.03)	.238
BMI (kg/m^2^)	25.7 (2.3)	29.3 (3.1)	.187
SBP (mm Hg)	128.0 (3.0)	135.0 (4.0)	.091
DBP (mm Hg)	73.0 (2.0)	77.0 (3.0)	.147

Data are expressed as mean (SEM); BMI: body mass index; WHR: waist-to-hip ratio; SBP: systolic blood pressure; DBP: diastolic blood pressure.

**Table 2:** Metabolic, lipid, and hormonal profiles in women with polycystic ovary syndrome and healthy controls.

Variables	Control (n=40)	PCOS (n=50)
Fasting plasma glucose (mg/dL)	77.3 (3.2)	82.0 (3.6) ns
Fasting insulin (pIU/mL)	14.2 (1.2)	25.3 (1.3)^a^
Glucose/insulin ratio	4.9 (0.22)	3.3 (0.13)^a^
HOMA-IR	2.7 (0.11)	5.1 (0.14)^a^
Triglycerides (mg/dL)	70.6 (5.3)	117 (6.4)^a^
Total cholesterol (mg/dL)	170.3 (8.0)	198.2 (6.0)^a^
HDL-C (mg/dL)	46.5 (2.2)	40.3 (1.4)^a^
LDL-C (mg/dL)	100.6 (4.1)	135 (4.3)^a^
VLDL-C (mg/dL)	21.4 (1.0)	26.6 (1.1)^a^
Luteinizing hormone (IU/L)	5.6 (1.02)	9.8 (1.05)^a^
Follicle stimulating hormone (IU/L)	6.12 (1.3)	4.7 (1.2) ns
Luteinizing hormone/Follicle stimulating hormone ratio	0.91 (0.11)	2.08 (0.14)^a^
Testosterone (ng/dL)	33.2 (3.1)	76.9 (5.3)^a^
SHBG (nmol/L)	55.3 (2.8)	40.2 (2.6)^a^
Free androgen index (%)	2.08 (0.12)	6.63 (1.3)^a^
17-OHP (mg/dL)	2.1 (0.13)	3.2 (0.11)^a^

Data are expressed as means (SEM).

PCOS: polycystic ovary syndrome; HDL: high high density lipoprotein; LDL: low low density lipoprotein; VLDL: very very low density lipoprotein;

SHBG: Sex hormone-binding globulin; 17-OHP: 17-hydroxy progesterone.

HOMA-IR: [glucose (in mg/dL×0.05551)×insulin (in μmIU/mL)l]/22.5; FAI=testosterone (nmol/L)/SHBG (nmol/L)×100.

a Significantly different from healthy control (*P*< .01); ns: non-significant.

## DISCUSSION

The present study shows that circulating ADMA, tHcy, hsCRP, Lp(a), and fibrinogen were higher in women with PCOS than in healthy controls. Our study confirms a positive association between these parameters and both IR and free androgen index. These findings are consistent with the results of several recent investigations.^2^,^7^,^19^ Hyperinsulinemia is a predictor of coronary artery disease, and IR has been proposed as a key factor linking linked to hypertension, glucose intolerance, obesity, lipid abnormalities, and coronary heart disease.^4^ IR may, in part, contribute indirectly to cardiovascular risk in PCOS by amplifying androgen excess. As a consequence of IR, PCOS patients often have an abnormal lipid profile and an increased incidence of cardiovascular risk factors.^6^ High levels of total cholesterol and LDL-C, and low levels of HDL-C are the most frequent forms of dyslipidemia, observed in insulin resistance states. Lower HDL-C level is another independent of risk factor for CVD associated with PCOS.^20^ As expected, in our study, we observed a significant increase in total cholesterol, LDL-C, and TG and a reduction in HDL-C in women with PCOS. Our results are in accordance with recent studies by Macut et al^19^ and Valkenburg et al^20^ who reported that lipid profile parameters were increased in patients with PCOS, suggesting that women with PCOS have a higher risk for CVD.

The results of the current study suggest that hyperandrogenemia in patients with PCOS has the strongest association with the presence of IR. This finding is supported by several other studies.^19^,^21^ IR, with elevated circulating insulin levels, induces unfavorable changes in lipid metabolism and increased androgen production from theca cells.^22^ At the same time, androgen excess may support the presence of an unfavorable metabolic state and lead to dyslipidemia. The mechanism by which hyperandrogenemia might affect vascular reactivity is still unknown. In experimental models, testosterone influences vasocontractile responses, and impairs endothelium-dependent relaxation in hypercholesterolemic rabbits and monkeys.^22^,^23^ Moreover, androgens may act synergistically with IR and inflammatory cytokines on vascular endothelial function.^1^

Elevated hsCRP was reported as a proinflammatory agent and a risk factor for the CV events.^14^ The present study demonstrated that circulating hsCRP levels were higher in women with PCOS compared with controls. Our results are in agreement with those reported by Heutling et al^24^ who documented higher hsCRP in women with PCOS than in healthy controls. Our data strengthen the idea that there is an increased risk of CVD events in women with PCOS.

Like other risk factors of CVD, hyperhomo-cysteinemia is associated with endothelial dysfunction, an early and reversible event of atherogenesis. Endothelial dysfunction results in reduced nitric oxide bioavailability and increased lipid peroxidation and the formation of atherogenic oxidized low density lipoprotein.^25^ High homocysteine levels are a risk factor for CVD because of the increased oxidative stress in the vascular endothelium and activation of platelet aggregation.^26^ In the present study, women with PCOS had significantly higher tHcy and lower nitric oxide levels, compared controls. Our data are in agreement with those reported by Badawy et al^27^ who also documented higher tHcy concentrations in women with PCOS.

In the present study, the plasma concentration of ADMA was significantly higher in women with PCOS compared with controls. The possible mechanisms of elevation of ADMA in hyperhomocysteinemia include reduced renal excretion^28^ and decreased activity of the hydrolase enzyme, which metabolizes ADMA.^29^

The correlations found in this study between nitric oxide bioavailability, hyperhomocysteinemia, chronic inflammation, IR, and hyperandrogenemia support the concept of interplay among these agents on the vascular bed in the pathophysiology of CVD in women with PCOS. Therefore, since impaired nitric oxide bioavailability is one of the first steps in atherogenesis, further evaluation of ADMA, tHcy, Lp(a), and hsCRP as potential markers in this these high-risk patients may have clinical relevance.

In conclusion, plasma ADMA, tHcy, Lp(a), and fibrinogen concentrations were elevated in Saudi women with PCOS compared to healthy women. Plasma nitric oxide and HDL-cholesterol were not significantly different between the two groups. This finding highlights the clinical importance of the assessment of the above mentioned markers in women with PCOS to reduce the risk of CVD.
